# Modeling of Neuronal Growth *In Vitro*: Comparison of Simulation Tools NETMORPH and CX3D

**DOI:** 10.1155/2011/616382

**Published:** 2011-01-20

**Authors:** J Aćimović, T Mäki-Marttunen, R Havela, H Teppola, M-L Linne

**Affiliations:** 1 Department of Signal Processing, Tampere University of Technology, P.O. Box 553, 33101 Tampere, Finland; 2 Department of Mathematics, Tampere University of Technology, P.O. Box 553, 33101 Tampere, Finland

## Abstract

We simulate the growth of neuronal networks using the two recently published tools, NETMORPH and CX3D. The goals of the work are (1) to examine and compare the simulation tools, (2) to construct a model of growth of neocortical cultures, and (3) to characterize the changes in network connectivity during growth, using standard graph theoretic methods. Parameters for the neocortical culture are chosen after consulting both the experimental and the computational work presented in the literature. The first (three) weeks in culture are known to be a time of development of extensive dendritic and axonal arbors and establishment of synaptic connections between the neurons. We simulate the growth of networks from day 1 to day 21. It is shown that for the properly selected parameters, the simulators can reproduce the experimentally obtained connectivity. The selected graph theoretic methods can capture the structural changes during growth.

## 1. Introduction

Development of computational tools has been one of the central topics in the computational neuroscience community. Several simulators of bioelectrical activity are publicly available and considered well-established tools. Both the cellular mechanisms behind this activity and the communication between cells, through the exchange of activity, can be modeled and analyzed using these tools [1]. In addition to the bioelectrical activity, the morphological structure of neurons and neuronal networks can be reconstructed by methods based on the experimentally verified morphological constraints [2]. Recently, two simulators were proposed, aiming to reproduce the morphological and structural changes of neuronal networks during growth [3, 4]. These two tools reproduce the morphological characteristics of neurons in each step of growth and not only in its final phase. Both provide a set of components that can be combined in a user-defined model, including functions defining axonal and dendritic growth, morphology of different types of neurons, or environmental constraints. They can reproduce growth in planar and three-dimensional space. Currently, they simulate solely the morphological aspects of neuronal circuits, but they will likely be extended, in the near future, to include the development of bioelectrical activity.

Various aspects of growth in neuronal systems can be analyzed using models [5]. Some models concentrate on details of biophysical processes related to one phenomenon, while others describe several processes with less details [6, 7]. Examples of analyzed phenomena are initialization of dendritic and axonal arbors [5], dynamics of intracellular chemicals involved in axonal and dendritic outgrowth [6], and selection of axon growth direction following guidance cues in the environment [8, 9]. The framework for phenomenological modeling of growth is proposed in [3, 10, 11]. Here, the statistics of morphological changes, including branching and elongation, are computed without a reference to the intracellular or extracellular processes leading to those changes. Finally, the models of growth of neuronal populations and formation of networks are proposed in [9, 12]. In [9], the influence of guidance cues on axonal growth and the developed network properties are studied. In [12], a study of activity-dependent growth of neuronal networks is presented. Recently, an activity-dependent model of growth was utilized to examine the self-tuning to criticality [13].

In this work, we assess the applicability of NETMORPH and CX3D simulators, proposed in [3, 4], to study the growth in neocortical cultures. The neocortical cultures represent an experimental model of moderate size neocortical circuits, consisting of 10,000–100,000 neurons. The immature neurons are extracted from the neocortex of rat embryos and plated on a dish. Such neurons, regularly supplied with nutrients, develop their full morphology and functionality. The proportion of different neuronal subtypes observed in cultures is similar as in the neocortex [14]. The neurons retain similar morphological and functional properties as those found in the neocortex [14–17]. Still, the cultures contain only a moderate number of cells which live in a suboptimal environment. Their organization is substantially different from the one observed in the neocortical tissue, since neurons form planar networks different from the layered three-dimensional columns in the neocortex.

Several aspects make cultures a valuable tool for the study of cortical circuits. In cultures, the study of relation between morphology and functionality is feasible, since both can be studied simultaneously using the established experimental techniques. The morphological changes can be followed using a combination of cell staining and microscopy [15–17]. At the same time, their functionality can be assessed using either the patch-clamp recordings from single neurons [18] or by recording the activity from several locations in the network using the microelectrode arrays (MEA) [14, 19]. The morphological and functional properties of neurons can be manipulated by changing the content of the extracellular space. Properly maintained cultures can survive for several months [14, 19], and all of the described methods can be applied at different times during the culture life which provides an experimental framework for study of growth. The simulated model of neocortical cultures is constructed using the information available in the literature. We opted for a model based on the statistical description of morphological changes during growth, proposed in [10, 11], without trying to model intracellular or extracellular biophysical processes. These models are the intrinsic part of NETMORPH simulator and the implementation of fundamentally different growth rules would require changing the core of the simulator. The CX3D allows more flexibility in model description, and the adopted growth rules are implemented following [3, 10, 11]. The considered model allows the precise reconstruction of single neuron morphology and the constraints it imposes on the connectivity between neurons. Still, the model employs relatively simple rules and does not depend on many parameters. Therefore, it is suitable for the analysis of structural changes in neuronal networks during growth. The implemented model does not consider the role of activity that spontaneously emerges in neuronal cultures, but it mimics the synapse formation. Potential synapses are formed whenever the presynaptic and the postsynaptic sites are close enough. A certain percentage of these synapses can be considered "functional". According to [20], the percentage of potential synapses that can be considered functional is 25% *in vivo*. This percentage might be significantly different in cultures, due to the absence of columnar organization, smaller number of neurons that form synapses, and smaller density of dendrites and axons. The experimental studies estimate that in a culture a neuron directly connects to 10–30 of other neurons through functional synapses [14, 18].

Section 2 presents the methodology used in this work. A description of simulators is given in Section 2.1. The implemented model is described in detail in Section 2.2. Section 2.3 describes the graph theoretical measures of connectivity of network structure. Section 3 describes the obtained results and conclusions. In Section 3.1, we discuss the properties of the two simulators from the user point of view. In Section 3.2, the number of synapses per cell and per dish is evaluated, while varying model parameters and the obtained statistics are compared to the experimental evidences from the literature. Section 3.3 shows the basic statistics evaluated on network graph, extracted from the simulation results at different development days. Finally, Section 4 gives the discussion of the obtained results and their implications in study of morphological changes during growth of neocortical cultures *in vitro.*

## 2. Material and Methods

In this section, the adopted model of neuronal growth in neocortical cultures is presented in detail, together with the implementation details in the two simulators. In addition, the measures used to assess the simulation outcomes to evaluate the two simulators are listed. 

### 2.1. Simulators of Growth

Recently, two simulators that can reproduce growth and development of neuronal systems were published in [3, 4]. Although both aim at simulating growth and development of neurons and neuronal networks, the methodology, implementation, and set of problems where they can be employed differ significantly. From the user point of view, the difficulty of model implementation, control of the parameters of the implemented model, and efficiency of simulations are also very different when using the two tools.

The NETMORPH simulator (http://netmorph.org/) has been developed at the Department of Experimental Neurophysiology at VU University Amsterdam. It is based on the extensive work on mathematical models that describe the morphology of dendritic and axonal arbors and the changes in the morphology during growth [7, 10, 11]. The software was written in C++ and provides a set of ready-made model components. The user can select the components and define the model parameters in a textual file called from the command line or directly from the command line. The software provides separate simulators for 2D and for 3D models which use the same set of model components.

The CX3D (http://www.ini.uzh.ch/~amw/seco/cx3d/) has been developed at the Institute of Neuroinformatics of the University of Zurich and ETH Zurich. The simulator includes a wide range of phenomena related to growth and development. Although we examine it in the context of growth in cultures, it can be employed to test many other processes including cell migration, formation of cortical columns, cell mitosis and apoptosis, and axon guidance through extracellular cues. The simulator is implemented in Java and the users are expected to write their own classes defining the model components and combining them into the models of neuronal systems. The simulator essentially represents three-dimensional systems, but it can be adapted for the simulation of cultures.

We used the versions of the simulators that were available on the listed web sites in January 2010. The software was tested on Linux and Unix servers and on the personal computers (4 GB RAM, 3 GHz processor) working under Windows or under MacOS. Both simulators can be straightforwardly installed and run under Linux and MacOS. The CX3D can easily be installed and used under Windows, while NETMORPH needs the Cygwin environment on Windows. Simulation time did not depend significantly on the choice of operating system when working on computers of comparable performances. 

### 2.2. Model of Neocortical Culture

**Figure 1:**
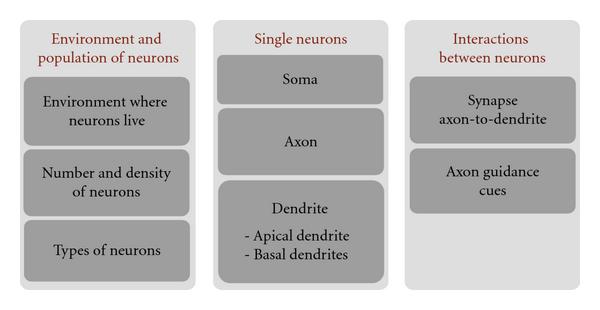
**Schematic representation of a model of neocortical culture**. Each block in the figure represents a set of related model components and model parameters that were selected from the literature.

**Figure 2:**
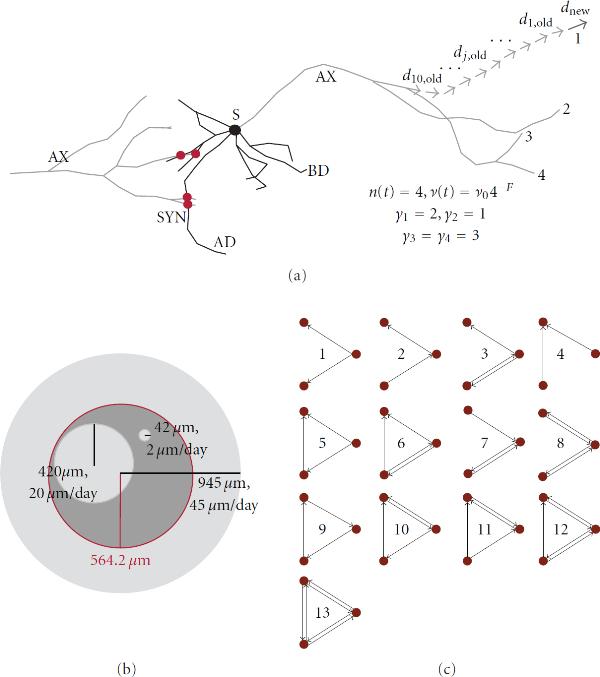
**(a) shows one pyramidal neuron (simulation in CX3D) and its soma (S), axon (AX), basal dendrite (BD), and apical dendrite (AD)**. In order to show synapse formation, axon of a proximal neuron is also shown. The synapses (SYN) are marked with red circles on the figure. The right side of the panel shows computation of the elongation rate (), centrifugal order for four terminal tips (, where ), and the new growth direction () from the direction of ten preceding segments. (b) illustrates the influence of elongation rate on the synapse formation. For smaller elongation rates, for example, 2 *μ*m/day, the maximal space covered by dendrites (or axons) at the end of a simulation is small and neurons cannot reach each other easily. For the larger values, for example, 20 *μ*m/day, every neuron can cover a substantial part of the dish and other neurons. For the large values, for example, 45 *μ*m/day, the dendrites extend beyond the dish space and are able to reach every one of 100 neurons. (c) illustrates all possible connectivity patterns between three neurons. These patterns are called *motifs*, and their frequency in a network represents the structural properties of that network.

In the presented study, we focus on growth in neocortical cultures. The models describing this phenomenon can be schematically represented as in Figure 1. The leftmost block defines the context, the characteristics of the environment where neurons live, and the characteristics of modeled neuronal population. The central block describes the model on single neuron level, for each implemented type of neuron. The rightmost block defines the interactions between neurons. Details of the model are illustrated on Figure 2(a), including the explanation of neuron anatomy and the meaning of the model parameters.

*The environment* block describes the shape and size of the space occupied by the neurons. In our model, this is a circular two dimensional dish in NETMORPH implementation, and a very flat cylinder, with the height equal to a cell diameter, in CX3D implementation. The CX3D models cannot be two dimensional due to properties of the simulator, but can be made nearly two dimensional by constraining the range of *z* coordinates to very small values. The dish radius is computed based on the number and density of neurons. In addition, the environment might define the properties of the medium that neurons live in, and the presence of particular chemicals that may influence growth. We do not model such extracellular chemicals explicitly, but the assumptions regarding the medium reflect on the choice of model parameters. It is assumed that neurons grow in an environment similar to the standard culturing medium, for example, as in [19]. The parameters are chosen to correspond to these conditions as well as possible.

*The number and density of neurons* are chosen as a tradeoff between the limitations of the simulators and the experimental evidence from the literature. The implemented networks consist of 100 neurons, which is the maximum number that can be simulated using both tools on standard personal computers. The selected density is 100 cells/mm^2^. The densities reported in the experimental literature vary from 300 cells/mm^2^ to 1380 cells/mm^2^ depending on preparation and the addressed question [18, 19, 21, 22]. The density in our model is lower than in the experimental studies to compensate for the intensive axonal and dendritic growth and the lack of apoptosis. The axons and dendrites grow simultaneously in the model, while in the experiments the beginning of axonal growth precedes the dendritic one [15]. The total number of neurons is fixed in the model, as a result of NETMORPH constraints, but in the experiments, the number of neurons starts to decrease after the second week *in vitro* [21]. The radius of the "dish" is computed to preserve the chosen density for 100 neurons. This gives the radius of 564.2 *μ*m. The "dish" determines the space where neuron somata are located, but the dendrites and axons are allowed to extend beyond its boundaries, in order to avoid boundary effect that would be severe for such a small radius. Effectively, we simulate a small portion of 0.1–1% of a neuronal culture.

We model the two most frequent *types of neurons* observed in cultures, the excitatory pyramidal neurons, and the inhibitory multipolar nonpyramidal neurons that correspond to the large GABAergic neurons reported in [17, 22]. The model culture consists of 80 pyramidal and 20 nonpyramidal neurons, following the usual proportion of excitatory and inhibitory neurons found in *in vivo* and *in vitro* [14]. An example of a pyramidal neuron, simulated using the CX3D, is shown on Figure 2(a). In addition, neuron soma (S), axon (AX), a basal dendrite (BD), and the apical dendrite (AD) are indicated on the figure.

*The description of soma* includes the soma size, its shape, and the number of dendrites extending from the soma. The diameter of the soma is fixed to 10 *μ*m for all the neurons following the experimental results from [21]. The somas are spheres or circles in the model, and their natural shape is mimicked using a particular placement of neurites on the soma. In pyramidal neurons, the apical dendrite is positioned opposite to the axon and the basal dendrites grow on the axon half of the soma. In the nonpyramidal neurons dendrites are placed randomly on the entire soma. The total number of dendrites in all neurons is between 4 and 6. In the case of pyramidal neurons, one of them is the apical dendrite, and the remaining 3–5 are the basal dendrites. 

*The models for axons and dendrites* are based on the statistical description of morphological changes during growth proposed in [7, 10, 11]. All the axons and dendrites use the same description with different parameter values. The parameter values are mainly obtained from NETMORPH tutorial paper [3]. The axon parameters are originally estimated from neurons growing in cultures and are available in [3]. The parameters for basal dendrites are taken from the three examples of reconstructed neurons *in vivo* available on NETMORPH web site. These parameters are consistent for the three examples, and we assumed that they grow similarly *in vitro*, since they are relatively short and placed around the soma and do not depend much on the guidance cues that exist *in vivo* but not *in vitro*. The parameters for apical dendrites are not available in the literature, and they evidently have different properties than those observed *in vivo* [15]. We assumed that they grow similarly to basal dendrites and used the same model, except that we adopted two times bigger initial elongation rate, following the microscopy images from [15]. The dendrites of nonpyramidal neurons are selected from the example of reconstructed basket cells, also available in [3] and on the web site. The motivation for this choice is found in [17], where large GABAergic neurons are studied *in vitro*. The study suggested that these neurons may be similar to the basket cells found *in vivo*. All the relevant model parameters are listed in Table 1.

**Table 1:** The list of the relevant model parameters. Model parameters are listed in the third column. The references used to obtain these parameters are given in the last column. Reference [3] points to the parameters that can be found directly in NETMORPH tutorial paper, while [3]* points to the reconstructed neurons from the same paper, which parameters can be found on the simulator web site. The notation "simulators" points to the value selected as a result of simulator constraints. The parameters for apical and basal dendrites differ only for  and they are shown together in the table (the  for the apical dendrites is given between the brackets). The initial elongation rates , marked with +, are varied in the simulations.

	Parameter	Value	Unit	Selection criteria
	Number of neurons	100		Simulators
	Proportion pyr.	80		[14]
	Proportion nonpyr.	20		[14]
	Density of neurons	100	cells/mm^2^	Simulators, [14, 18, 19, 22]
	Soma diameter	10	*μ*m	[21]

Axon		45	*μ*m/day	[3]
		0.16		[3]
		17.38		[3]
		14	days	[3]
		0.39		[3]
		0		[3]

Basal (apical) dendrite		9.635 (19.27)	*μ*m/day	[3]* ([3]*, [15])
		0		[3]*
		2.52		[3]*
		3.006	days	[3]*
		0.73		[3]*
		0.5		[3]*

Nonpyr. dendrite		9.635	*μ*m/day	[3]*, [17]
		0		[3]*, [17]
		2.6475		[3]*, [17]
		4.706	days	[3]*, [17]
		0.594		[3]*, [17]
		−0.259		[3]*, [17]

Synapses NETMORPH	Distance pyr.-pyr.	1	*μ*m	[3]
	Distance pyr.-nonpyr.	0.1	*μ*m	[3]
	Distance nonpyr.-pyr.	1	*μ*m	[3]
	Distance nonpyr.-nonpyr.	0.1	*μ*m	[3]

Synapse CX3D	Spine length	3	*μ*m	[4], [23]
	Bouton length	2	*μ*m	[4]

The model for every axon or dendrite consists of three components: elongation of the terminal segments, branching of the terminal segments, and the model defining the shape of terminal segments and, consequently, the shape of segments between successive branching points. These components are also illustrated on Figure 2(a). The CX3D is a general purpose simulator, and various models of growth can be implemented and tested. On the contrary, NETMORPH focuses on a particular set of models that reproduce the statistics of axonal and dendritic morphology but do not tackle the biophysical mechanisms leading to this statistics. Therefore, the model is mainly constrained by NETMORPH, which, also, makes it more adapted to this simulator.

The elongation of terminal segments depends on the initial elongation rate  and on the current number of terminal segments in the same arbor  and is given by (1), described in details in [3]. The dependency on the number of terminal segments is regulated by the parameters . The terminal segments in the same arbor elongate with the same speed at a given time. Although they can vary for small random values, this choice made CX3D implementation simpler. To illustrate, the elongation rate is computed for an axon with 4 terminal segments and shown on Figure 2(1)

The branching of terminal segments is defined by the probability of branching, the initial length assigned to the two new segments, and the initial angle between them. The probability of branching, , for the terminal segment with index  is given by (2), described in details in [3]. It depends on the expected number of branchings in the arbor, , on the total number of terminal segments at the considered time instant , and on the centrifugal order of the considered terminal segment , . This last parameter is equal to the number of branching points between the considered terminal tip and the root of the arbor it belongs to. Figure 2 illustrates computation of circular order for the 4 terminal segments of the axon. The remaining model parameters are the time constant , dependency on the number of terminal tips , and dependency on the centrifugal order . The normalizing coefficient is denoted as . The parameter  indicates the time step used to simulate the model(2)

The initial length of new segments depends on the simulator. In NETMORPH, they are selected randomly by dividing the last elongated part of the terminal segment. In CX3D, they are controlled by the segment "default length" set to 10 *μ*m. The choice of the initial length affects the overall simulation result less in CX3D than in NETMORPH. The first one implements the tensions in a segment during elongation and retraction, as well as the mechanical interaction between different segments and different neurons. This intrinsic model dynamics modulates the length of the developed axons and dendrites. The initial angle between each new pair of segments is  in both simulators, since it is fixed to that value in CX3D.

The shape of the axons and dendrites is determined by the model component that defines growth direction in each time step. In NETMORPH, the shape is entirely defined by the sequence of movements directions of the terminal segment. It is computed as the weighted sum of the directions of previous segments, taking into account the segments up to the last branching point. The new direction is defined as . Here,  is the length of the considered segment ,  is the distance between its center and the tip of the terminal segment (computed along the segments),  are direction vectors, and  is a random perturbation. Computation of the new direction vector  from the set of previous direction vectors  is shown on Figure 2 for one terminal segment of the axon. The probability that direction changes is equal to , where  is the elongation during the last time step. In CX3D, the shape is determined dynamically. The movement direction in each time step is initially computed as the random perturbation of the direction from the previous time step as . Still, the final movement direction depends on the internal and external forces that affect the segment. The internal mechanical tensions in the segment determine how much it can be elongated or retracted. The segments tend to avoid obstacles which lead to changes in direction and bending of the segments. We also implemented NETMORPH growth direction model in CX3D, but the obtained result did not seem to represent the growth better than the original choice.

*The model for synapse formation* is based on the proximity criteria between pairs of axons and dendrites. Still the implementation of the model significantly differs in the two simulators. The NETMORPH requires a user-defined maximal distance between the presynaptic and the postsynaptic sites. For each axon-dendrite pair being on a distance smaller than the maximal, a synapse can be created with the likelihood inversely proportional to the distance. The maximal distances can be defined for different types on neurons and are listed in the Table 1. In CX3D, the actual growth of axonal boutons and dendritic spines is simulated. The length of the formed boutons and spines corresponds to the experimentally observed values. If a spine and a bouton touch each other, they form a synapse with certain probability than can be specified by the user. The model for synapse formation is the same for all neuron types, a choice imposed by the simulator. Figure 2 illustrates formation of four synapses (red circles) on the contacts between the dendrites of the depicted neuron, and the axon of another proximal neuron (not shown on the figure).

*The axon guidance cues* are the chemical species in the extracellular space that influence the axon growth directions when sensed by its tip. Both simulators allow some possibility to model the guidance cues, but only CX3D allows the simulation of chemical diffusion in the space. We opted to leave this mechanism out of the presented study and consider its influence only in the future work. 

#### Parameters Varied in Simulations

In order to test the network formation in model cultures, we varied a parameter that significantly influences the growth and synapse formation. The probability of synapse formation is determined by the capability of neurons to reach each other, which is controlled by the elongation rate of axonal and dendritic trees. Figure 2 illustrates the maximal portion of the space covered by basal dendrites of a single neuron for different elongation rates. Four different elongation rates can be varied in the model: for axons, for apical and basal dendrites of pyramidal neurons, and for the dendrites of nonpyramidal neurons. In the simulations, the initial elongation rate  is varied for basal dendrites. The same parameter for axons, apical dendrites, and dendrites in nonpyramidal neurons is chosen as the value for basal dendrites multiplied by 4.5, 2, and 1, respectively. The examined values for the elongation rate of basal dendrites are 1, 2, 4, 6, and 8 *μ*m/day in NETMORPH and 2, 6, 10, 14, and 22 *μ*m/day in CX3D. Axons and dendrites in CX3D are elongating slower due to their internal dynamics, and somewhat bigger values for  were needed to obtain the comparable results to NETMORPH.

### 2.3. Statistical Measures of Graph Properties

The neuronal networks developed until days 4, 7, 10, 14, and 21 are extracted and converted into unweighted directed graphs. Every neuron represents a node in the graph, and every synapse between two neurons introduces an edge between the two corresponding nodes. Multiple synapses between the same pair of neurons are not considered. Denote the set of nodes as  and the set of edges as . For the obtained graphs, properties such as in- and out-degree distribution, shortest path length, and the frequency of motifs are computed.

The in-degree of a node is computed as the number of edges arriving at the node, that is, the number of postsynaptic contacts of the corresponding neuron. The out-degree of a node is the number of edges leaving the node, that is, the number of presynaptic contacts of the corresponding neuron.

For every pair of nodes,  and , the number of edges in every path connecting these nodes is computed. The smallest number of edges determines the shortest path. The distribution of shortest paths for all the pairs of nodes in the network measures the global connectivity in that network. It indicates how well the nodes are connected and how fast information can be transmitted within the network.

The count of motifs [24, 25] is a measure of local connectivity. It indicates how well the neighbors connect, how pronounced the clustering in a network is, and how often the nodes form loops. All triplets of nodes in the network are examined in order to count different connectivity patterns. Such patterns between triplets of nodes are called motifs, and there are, in total, 13 different motifs (shown in Figure 2 and in [24, 25]). The frequency of different motifs is considered to be an indication of distinct structural properties [25]. 

## 3. Results

This section summarizes the results and conclusions obtained by simulating the described model of growth using the two tools, NETMORPH and CX3D. A network of 100 neurons was constructed using the parameter set described in Table 1 and Section 2.2. Some of the parameters were varied, namely, the initial elongation rate () for axons and all types of dendrites. The proportion between the elongation rates of axons, basal, apical, and nonpyramidal dendrites is fixed and only the overall intensity of growth influencing all of them is varied. Five different parameter sets were tested for each of the simulators. The simulations reproduce the growth of neurons from the first day after plating them on a dish until the end of the third week (day 21) on the dish. The simulation step size was fixed to 0.1 h, a value sufficiently small to ensure stable simulations with both tools. 

### 3.1. Computational Efficiency

The efficiency of the tested simulators was considerably different. In NETMORPH, the execution of one batch simulation consisting of 120 repetitions for five sets of parameters required between 2 hours and 7 days depending on the choice of model parameters. In CX3D, the same simulation required between 4 and 40 hours for a single repetition and a single set of parameters. Therefore, collecting 120 repetitions for five parameter sets would require several weeks. From the simulation efficiency point of view, NETMORPH was evidently superior to CX3D. It should be pointed out that we selected the model adjusted to NETMORPH, so the differences in performance are not surprising. In CX3D, the limiting factor that influences the efficiency is the internal dynamics associated to every model element, that is, soma and neurite segment. It is created to mimic the natural interactions between model elements, but it requires memory space and computational time. The purpose of CX3D simulator is to provide a basis for modeling and analysis of virtually unlimited set of problems. The aim of the developers was to propose a sufficiently efficient general purpose tool, which might be suboptimal when focusing on one single class of models like in this study. 

### 3.2. Dependence of Synapse Density on Model Parameters

**Figure 3:**
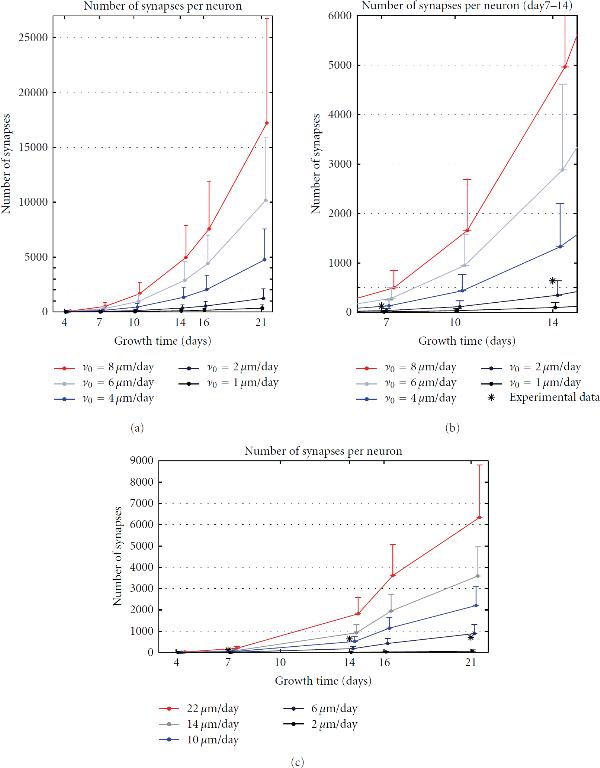
**Synapse density.** The upper row gives results for NETMORPH and the bottom row for CX3D. The curves mark the mean values, and the bars show the standard deviations. (a) shows the mean number of synapses per neuron when the elongation rate for basal dendrites takes values 1, 2, 4, 6, and 8 *μ*m/day. (b) shows the magnified region of interest from (a), that is, the interval between 7 and 14 developmental days. The "*" mark the experimental values for the corresponding days, taken from [21]. (c) shows the synapse density obtained using CX3D, and the elongation rates for basal dendrites equal to 2, 6, 10, 14, and 22 *μ*m/day. The experimental data (*) correspond well to the values obtained for  *μ*m/day.

The first set of simulations, summarized in Figure 3, was used to test the simulator and model properties. We focused on how well the simulators and models reproduce the synapse formation. The results obtained from the two simulators were compared with the corresponding experimental results found in the literature [21].

The number of postsynaptic and presynaptic sites, that is, the number of inputs and outputs, was computed for every neuron in every simulation repetition (120 repetitions in NETMORPH, 50 in CX3D). For each set of parameters, the mean and standard deviation were computed from the values obtained for 100 neurons and all the repetitions. Figures 3(a) and 3(b) show the results obtained for NETMORPH, and the bottom panel the results for CX3D. The curves on the panels connect the mean values obtained for days 4, 7, 10, 14, 16, and 21. The standard deviations are indicated with the one-side bars attached to the curves. The five curves, from blue to red, correspond to the five different values for the initial elongation rates. The chosen initial elongation rates for the basal dendrites of pyramidal neurons are indicated in the figure (see legend). For NETMORPH these values are , 2, 4, 6, and 8 *μ*m/day, and for CX3D, they are , 6, 10, 14, and 22 *μ*m/day. For the axons, apical dendrites, and dendrites of nonpyramidal neurons, the initial elongation rates are set to , , , respectively. Figure 3(b) is a magnification of the region of interest from Figure 3(a), that is, for days 7–14 which represents the most accurately simulated phase of growth using the described model. Before day 7, the synapse formation is affected by timing of axonal and dendritic growth. It has been shown that axonal growth precedes the dendritic one [15]. This aspect of growth cannot be included in our simulation, due to the NETMORPH constraints. After day 14, the overall synapse density decreases due to the pronounced apoptosis in cultures [21]. This is, also, excluded from our model that has a fixed number of neurons. 

Figures 3(a) and 3(b), obtained for NETMORPH, indicate an exponential increase in number of synapses per neuron over time. As expected, these numbers also increase when increasing the initial elongation rate. On average, increasing the elongation rate by 1 to 2 *μ*m/day increases the number of synapses 2-3 times for the same day of growth. All of the obtained values are significantly higher than the experimental results shown in [21]. The reported experimental values, computed as the total number of synapses divided by the total number of neurons, are around 64 synapses per neuron at day 7, 319 at day 14, 355 at day 21, and 1130 at day 28 [21]. In Figure 3(b), the double values of these experimental data for days 7 and 14 are marked with "*". The values are doubled, since we consider every synapse twice, once for the presynaptic and once for the postsynaptic neuron. The density computed in [21] "assigns" every synapse to one neuron although it belongs to the two neurons. These values fall between the simulation results obtained for  *μ*m/day and  *μ*m/day. Regarding the increase in the number of synapses between days 7 and 14, it most likely resembles the curve for  *μ*m/day. The high number of synapses may be explained by the tendency of the NETMORPH simulator to produce many synapses between the same pair of neurons.

Figure 3(c), obtained for CX3D, shows much better agreement with the experimental results. The increase in synapse number is not so dramatic as in NETMORPH, and the maximal values stay in the range of a couple of thousands. The differences obtained for different elongation rates are not so big as in NETMORPH. Finally, the simulation results obtained for  *μ*m/day show very good agreement with the experimental values for days 7 and 14. 

### 3.3. Statistics of the Network Graphs

**Figure 4:**
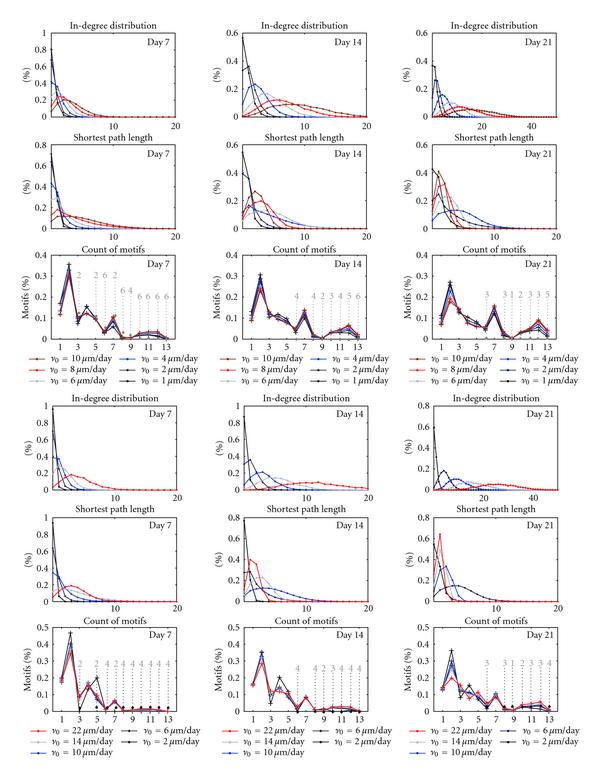
**Structural changes of the growing networks: in-degree distribution, shortest path length, and the count of motifs**. Three upper rows: NETMORPH results, three lower rows: CX3D results. Different curves correspond to different initial elongation rates , and the employed values are given in the legends (1, 2, 3, 6, 8, and 10 *μ*m/day for NETMORPH, 2, 6, 10, 14, and 22 *μ*m/day for CX3D). Grey interrupted lines indicate motifs that are significantly more frequent than in random networks (-test, 0.01 significance level). The corresponding numbers show for which parameters this holds; for example, 2 means "holds only for the two smallest values." For CX3D,  *μ*m/day was excluded since it gives too sparse random networks.

The extracted networks obtained in different phases of growth are analyzed using graph theoretic measures. The results for both simulators are illustrated in Figure 4. The three upper rows show the statistics of in-degree distribution, shortest path length, and motifs count computed from the networks simulated in NETMORPH. The three bottom rows give these same measures evaluated for the networks simulated in CX3D. Each panel corresponds to one of days 7, 14, or 21. Different curves in the same panel show the results obtained for different values of the initial elongation rate , and the values of  used for the basal dendrites are indicated in the legends. The statistics for all NETMORPH results is computed for 100 neurons in each network, and for 120 repetitions of each condition. The number of repetitions for CX3D simulations was 50.

*The in-degree distribution* in all the panels shifts toward higher values during growth and is higher for bigger values of growth rate. These results can be compared to the experimentally estimated connectivity in cultures, shown to be in the interval of 10–30% [14, 18]. This indicates that the values  and 2 *μ*m/day give too small, while the  and 10 *μ*m/day result in too high connectivity. Taking into account the conclusions from Figure 3, the values  and 6 *μ*m/day may give the results closest to the desired ones. A similar observation holds for the networks simulated in CX3D. Here, the overall growth of the neurites is slower due to properties of the simulator, so we used somewhat higher values for the elongation rates. The smallest tested value also gives too sparse networks, while the highest overestimated the connectivity. In CX3D, the values 10 and 14 *μ*m/day give the connectivity closest to the expected. Similar results were observed for the out-degree distribution.

*The shortest path length* distribution depends on the selected initial elongation rates. The slowly growing networks ( *μ*m/day for NETMORPH,  *μ*m/day for CX3D) form a small number of connections until day 7. Most of the neurons are not connected or connected to a few neighbors. The shortest path is computed from this small set of short local connections, which results in a narrow distribution peaked around 0. As the network grows, new connections are established and distant pairs of neurons start to connect indirectly through other neurons. This shifts the shortest path length toward higher values. Neurons in the faster growing networks ( *μ*m/day for NETMORPH,  *μ*m/day for CX3D) already form direct and indirect connections at day 7. In the following days, new connections are added which continuously decreases the shortest path, since more neurons become directly connected.

*The motifs count* is shown as the percentage of total number of connected triplets of neurons (see Figure 4). The obtained counts are similar for all the parameter values, particularly in the NETMORPH examples. The peaks are visible for the motifs 2, 4, and 7. In the equivalent random networks, the motifs with two edges only (1, 2, and 4) or three edges (3, 5, 7, and 9) are the most frequent. Still, not all of them are equally represented in the simulated networks. In order to compare the simulation results with the corresponding random networks, that is, the networks with the same probability of connection, the statistical tests are done (-test, with 0.01 significance level). The results are also shown in Figure 4, where dashed gray lines indicate the motifs that are significantly more frequent in the networks simulated using NETMORPH or CX3D than in the random networks. The number above each line shows for how many parameter values this holds, assuming that these are the smallest values from the set. In other words, number 4 indicates that a certain motif appears significantly more often in the networks simulated for the four smallest values among all the tested values, and it is either significantly smaller or not significantly different for the bigger elongation rate values. In CX3D figures, the smallest elongation rate was not considered, since it often gave very sparse random networks where motifs comparison was not possible. Expectedly, the motifs with four or more edges appear much more often in the networks simulated using NETMORPH or CX3D. 

## 4. Discussion

### 4.1. Comparison of the Simulators

The presented results reveal several important differences between the two simulators, and indicate when each of them should be employed.

Our general conclusion is that NETMORPH, which implements computationally inexpensive models, could be more useful in theoretical studies and particularly for analysis of large networks. The NETMORPH models do not depend on many parameters, and the influence of each parameter can be carefully monitored. The main problem in the current version of the simulator is the excessive formation of synapses, which leads to unrealistically high number of synapses per neuron, and consequently, to very large output files. Such files are difficult to manipulate and analyze, which is particularly limiting when working with large networks. Recently, the authors of the simulator proposed an advanced algorithm for synapse formation [26] that might help to overcome this problem.

The principal advantage of CX3D is its flexibility. The authors aimed at constructing a multipurpose simulator of neuronal growth that can be used to model development of different neuronal systems, and include various relevant mechanisms. This simulator is valuable when modeling a small number of neurons equipped with intracellular and extracellular chemical species. It might be useful for constructing multilevel models that incorporate cellular and network levels, and in the future the level of genetic networks. On the other hand, when implementing systems of 100 or more neurons with axons and dendrites that branch extensively, this simulator led to slow and memory consuming simulations. The complex dynamics of model elements, which is an intrinsic part of this simulator, requires time-consuming computations in every time step. In addition, it limits the maximal simulation time step that can be safely employed. Although the implemented model mainly behaved according to expectations, from time to time unwanted outcomes emerged as a result of the boundaries imposed by the nearly two-dimensional geometry of the environment. It was possible to observe axons or dendrites growing in a tight zigzag pattern in the situation when those segments found themselves "imprisoned" between the boundaries of the two-dimensional space and the surrounding objects. Finally, the complexity of the simulator imposed many "hidden" variables that were difficult to control, and whose influence on the simulation results was not obvious. 

### 4.2. The Employed Model

We analyzed a phenomenological model established in the literature and adapted it to model the growth in neocortical cultures [3, 7, 10]. The model neither describes the role of the activity as in [12, 13], nor the biophysical processes governing growth as in [9], but it can reproduce the growth of axons and dendrites and can be used to study the network formation. In addition, it is relatively simple and depends on a small number of parameters.

The complete list of model parameters is given in Table 1. The parameters that define population of neurons, like number and density of neurons, or percentage of pyramidal and nonpyramidal neurons, are either well established knowledge in the literature or imposed by the simulator constraints. The density can be chosen more freely, but, when focusing on synapse formation, varying the density is equivalent to varying the elongation rate, since both parameters determine how fast pairs of proximal neurons can reach each other. The synapse formation is determined by the maximal distance between pairs of neurons that can form a contact. In NETMORPH, it is set to very small values, and increasing them leads to even bigger production of synapses. In CX3D, these parameters are set according to the well known estimation of dendritic spines and axonal boutons length. Increasing the parameters would lead to unrealistic synapse formation model. Finally, the set of parameters that defines branching and elongation of dendrites and axons is the most interesting to test. In total, the model has six parameters for each of the four types of arbors, axons, basal and apical dendrites of pyramidal neurons, and basal dendrites of nonpyramidal neurons. Five of six parameters define the branching probability, which determines the overall shape of the arbor and also influence elongation. The elongation rate influences solely the size of the arbors and how far a neuron can "reach." Different neuron types have distinct morphology, and the branching rate parameters should be selected to provide the correct morphology. The elongation rates should also be chosen to reflect the size of axons and dendrites relative to each other. In this work, we opted to examine solely the influence of the elongation rate, the parameter that most directly affects neuron size and, consequently, the probability of forming synapses. This is also motivated by the choice of neuron models in [12, 13], where neurons are represented as circular fields without detailed morphology. The global connectivity measures, like in-degree distribution or shortest path length, are likely dominantly influenced by the elongation rate. This dependency is visible on the presented results. The local connectivity measures, like motifs frequency, might significantly depend on the local shape of axonal and dendritic arbors. The influence of branching rate parameters on the arbor shape and the frequency of motifs will be the subject of future studies.

Several adopted choices in the model can be reconsidered. For example, all of the neurons have the same model for axon, which might not be correct. The branching of the apical dendrite is not very precisely set although the elongation reflects the ratio between the growth of basal and apical dendrites of the same neuron [15]. The environment in which neurons live might affect the growth. Finally, the model for axon guidance is not considered here although it is known that it influences the growth of axons and their capacity to reach other neurons. Some of the listed criticisms can be corrected by fitting the model from the experimental data instead of using the parameters available in the literature. Such data can be collected using conventional methods, like the combination of staining and microscopy. Still, the available methodology requires time-consuming experiments in order to provide sufficiently rich data set for parameter fitting. 

### 4.3. Structural Changes During Growth, Graph Theoretic Study

Three measures were evaluated in order to analyze structural changes in networks during growth, in-degree distribution, shortest path length, and motifs count. Expectedly, since no criteria for stopping the growth were implemented, the number of synapses and the connections between neurons increased continuously. For the selected model parameters, the realistic values of in-degree and out-degree distributions, that correspond to experimental results, were obtained for days 7 to 14. The shortest path between a pair of neurons is initially small, with only a few connections established. As the network grows and new connections are added, the shortest path increases due to the new established pathways. When the number of direct connections between neurons increases enough, the average shortest path starts to decrease again. The motifs count reveals certain motifs that are more frequent in simulated than in the corresponding random networks for all the developmental days and majority of tested model parameters. These motifs have bi-directional connections. This might indicate that randomly placed neurons on the dish tend to find a proximal neuron and strongly connect to it by forming a loop.

The standard graph theoretic measures have been used in the literature to characterize mature cortical networks. Both small-scale networks of neurons and large-scale networks of cortical regions have been analyzed [27]. The statistics obtained *in vitro* cannot be straightforwardly related to the *in vivo* studies, since neurons develop outside of three-dimensional cortical columns and without the guidance cues present *in vivo*. In our study, the global measures of connectivity, like in-degree distribution and shortest path length, show expected results and can be used to indicate when the network becomes connected and which elongation rates give realistic results during the first two weeks of growth. The motifs count does not give straightforward result, but mainly indicates frequent bidirectional connections in the simulated growth models compared to the random networks. In this study, we analyzed the connectivity of unweighted graphs, assuming that neurons that are likely to connect form multiple synapses and sufficiently strong connections. Also, synapse pruning is not taken into account, a mechanism that would remove connections formed through small number of synapses. The study on weighted graphs, where each weight corresponds to the number of synapses between a pair of neurons, will give more accurate results. This work will be pursued in the future.

To conclude, in this study we constructed a phenomenological model of neuronal growth in cultures and tested it on the two recently published simulators of growth. The graphs extracted from the obtained networks were analyzed and the observations were compared to experimental results. Both simulators can reproduce the considered experimental values, but their overall behavior might be improved by implementing additional mechanisms. The analysis of the network structure revealed the expected structural changes during growth and formation of local motifs. 
